# Possible Link Between Tamoxifen and Suicidality: A Case Report Analysis of a 43-Year-Old Woman With Mood Disorder and Breast Cancer

**DOI:** 10.7759/cureus.69545

**Published:** 2024-09-16

**Authors:** Jaswin Singh, Noorvir Kaur, John Gurski

**Affiliations:** 1 Psychiatry, Reading Hospital Tower Health, West Reading, USA; 2 Psychiatry, Drexel University College of Medicine, Philadelphia, USA

**Keywords:** breast cancer, selective estrogen receptor modulator, suicidality, suicide, suicide prevention, tamoxifen

## Abstract

Tamoxifen, a selective estrogen receptor modulator (SERM), has long been a cornerstone in the treatment of hormone receptor-positive breast cancer. While its benefits in reducing the risk of recurrence and mortality in this patient population are well-established, emerging evidence suggests a potential association between tamoxifen use and adverse psychiatric effects, including suicidality. Despite extensive research on the clinical efficacy of tamoxifen, the exploration of its psychiatric side effects, including suicidality, remains relatively understudied. We present a case of a woman with a medical history of depression and invasive lobular carcinoma of the left breast who developed a severe episode of recurrent major depressive disorder (MDD) and attempted suicide with a benzodiazepine overdose upon initiating tamoxifen for her breast cancer. This case report aims to contribute to the growing body of literature on the relationship between tamoxifen and suicidality by presenting a detailed analysis of a patient who experienced suicidality while undergoing tamoxifen therapy.

## Introduction

Breast cancer remains a leading cause of mortality and is the most common type of cancer in women, with an estimated incidence of 1.6 million cases per year worldwide [1]. Tamoxifen is a first-generation selective estrogen receptor modulator (SERM) medication used to treat estrogen receptor (ER)-positive breast cancer in men and women and as a prophylactic agent against breast cancer in women [2]. In breast tissue, it specifically acts as a competitive antagonist with estrogen for binding sites and causes antiestrogenic and antitumor effects. It also slows cell cycling through downstream intracellular processes, classifying it as cytostatic [2]. It is often used in the treatment of female patients with ductal carcinoma in situ (non-invasive breast cancer) after surgery and radiation to reduce the risk of invasive breast cancer [2].

Clinical studies suggest that tamoxifen, along with its antiestrogenic effect, may reduce the postsynaptic response to serotonin and brain serotonin, and norepinephrine-related activity [3]. As tamoxifen can block the neuroprotective action of estrogen in the brain [3], there may be an association between tamoxifen treatment and a patient’s onset of severe depressive symptoms. Some large cohort studies exploring this issue have yielded similar results describing the onset or the exacerbation of depressive-type mood alterations while undergoing tamoxifen therapy [3]. 

An early review in 1999 brought attention to the potential link between tamoxifen use and depression, drawing from several studies indicating either an increased incidence of depressive symptoms compared to non-tamoxifen users or evidence of heightened depression following tamoxifen initiation [4]. A case-control study involving 42 postmenopausal women with breast cancer who received tamoxifen revealed statistically significant elevated depression scores [4]. A larger prospective trial (N = 257), in which mood symptoms in two groups of breast cancer patients were studied: those who received tamoxifen and those who did not. The study revealed that 15% of tamoxifen-treated patients experienced depression, compared to 3% of non-tamoxifen users, with a statistically significant difference noted. Overall, 31% of the patients experienced "significant depression," and 27% discontinued tamoxifen due to adverse effects [5]. 

Further literature review suggests conflicting evidence regarding the association of suicidality and tamoxifen, which includes a retrospective cohort study comparing patients with breast cancer, stratified by hormone receptor status and tamoxifen use, which did not find a statistically significant hazard ratio for "new-onset depression" among those receiving tamoxifen compared to those who were not receiving it [6]. Similarly, a multicenter randomized, placebo-controlled trial conducted by the National Surgical Adjuvant Breast and Bowel Project, which aimed to assess various health outcomes, including depression, did not identify a statistically significant association between tamoxifen use and depression [7]. 

Despite these findings, the absence of a statistically significant relationship in these studies does not preclude the possibility of an association, and further research is warranted to elucidate the potential link between tamoxifen use and suicidality comprehensively. Herein, we report a case of a 43-year-old woman who developed an episode of suicidality necessitating inpatient psychiatric treatment following the initiation of her tamoxifen treatment.

## Case presentation

The patient presented with a pertinent medical history of major depressive disorder (MDD), generalized anxiety disorder (GAD), and invasive lobular breast cancer and was seen in the emergency department following ingesting seven to eight tablets of 0.5 milligrams of Xanax (alprazolam) in a suicidal attempt. Her home psychiatric medications included Xanax 0.5 milligrams three times daily as needed for anxiety, buspirone 20 milligrams twice a day for anxiety, vortioxetine 20 milligrams nightly for depression, propranolol 80 milligrams daily for anxiety, and zolpidem 5 milligrams nightly for insomnia. She had been on this medication regimen prior to tamoxifen therapy and never experienced mood instability or severe suicidal ideations or attempts prior to this episode. 

During the psychiatric evaluation, she was noted to be cooperative on the exam. However, she had a behavioral disturbance in which she displaced her IV line. After redirecting and reorienting the patient, she endorsed symptoms of depressed mood, impulsivity, insomnia, and hopelessness. She denied symptoms of mania or psychosis. She described her mood at the time of evaluation as “depressed, anxious, and angry.” Her affect was noted to be mood-congruent. She was goal-directed without hallucinations, delusions, or paranoia. She denied active suicidal or homicidal ideations or thoughts at that time. Insight and judgment were rated as “good” by the evaluating psychiatrist. She was admitted to an inpatient psychiatric unit on a voluntary basis after daily evaluations in which the patient continued to be cooperative and did not have suicidal ideations. At this point, the evaluating psychiatrist deemed she was stable for discharge after 72 hours without any medication changes. 

On her outpatient follow-up, the patient endorsed believing her mood dysregulation was secondary to tamoxifen, which she had started one month prior to the suicide attempt. She noted feeling “angry all the time” and “snapping out” at her family, which began after tamoxifen therapy was initiated. She made the decision to proceed with the divorce from her husband, although their relationship was noted to be “loving” and “supportive.” She had self-discontinued tamoxifen and endorsed the resolution of her mood symptoms, denying further mood instability, suicide attempts, or insomnia. Her zolpidem was also discontinued, but the rest of her home medication regimen remained the same. On recurrent follow-up visits, the patient remained euthymic. 

## Discussion

Tamoxifen effectively acts to decrease estrogen output in the breast, thus decreasing tumor and cell growth [8-10]. This decrease in estrogen is believed to negatively impact mood, as it is well known that estrogen products also increase serotonin receptor density [10]. Estrogen receptors have been found in the amygdala, hippocampus, and hypothalamus as well. These regions have been shown to have increased neuroplasticity induced by estrogen [11]. Tamoxifen is also known to inhibit protein kinase C, a key enzyme implicated in neuroplasticity, and is also the target of mood-stabilizing medications like lithium and valproic acid [12]. Although a question of acute or chronic, it can be hypothesized that through decreased estrogen and thus neuroplasticity and receptor density, memory, mood, and regulatory functions can be negatively impacted [12]. 

The patient, who had a long-standing history of pre-existing MDD and GAD, never had a suicidal attempt or any self-injurious behaviors prior to starting tamoxifen. The presenting patient had endorsed at least two weeks of mood instability, leading to irritability and divorce in an otherwise amicable relationship with her husband. Similarly, case reports have shown sudden onset of mood-related issues shortly after starting therapy, much like this patient. There has been only one reported case of suicidal attempt via self-inflicted gunshot wound after starting tamoxifen therapy [8]. That patient’s presentation was consistent with bipolar II disorder after starting bupropion, which ultimately stabilized through treatment with lamotrigine [8]. 

Dopamine and serotonin are known to play major roles in mood regulation. Research has shown that serotonin is implicated in the inhibition of aggression. Multiple studies have supported the hypothesis that serotonin and dopamine have an inverse relationship, thus leading to an increase in impulsive behaviors [13]. This hormonal imbalance may have led to the patient’s impulsive attempt to ingest 3.5 grams of Xanax and new-onset thoughts to divorce her husband. There has also been an association between 5HTTLPR serotonin transporter gene polymorphism and violent suicidal behavior [13]. The patient’s mother also had a history of depression and anxiety, possibly predisposing the patient to lower serotonin metabolism via genetic factors. Given the new onset of impulsive behaviors, it could be hypothesized that there may be an interplay between physiological and genetic components.

In multiple cases, cessation of tamoxifen led to a relatively quick improvement in mood [8, 9, 10]. This highlights the importance of close psychiatric monitoring in patients with underlying mood disorders. In patients where tamoxifen may be the only viable therapeutic option, its effects may be mitigated by using antidepressants that would not affect tamoxifen metabolism. Such antidepressants include venlafaxine, which can help to stabilize mood and decrease impulsivity, as some antidepressants inhibit the metabolism of tamoxifen to its more active metabolites by the cytochrome P450 system [14]. 

## Conclusions

The risk of developing depressive symptoms or full-blown depressive episodes during tamoxifen use has been repeatedly raised. This report aims to emphasize the potential link between tamoxifen and suicidality. In doing so, we hope to promote the importance of vigilance among healthcare providers regarding the psychiatric well-being of patients receiving tamoxifen therapy, as well as the need for further research to characterize better and mitigate these risks. Understanding the interplay between tamoxifen and suicidality is paramount in optimizing the safety and efficacy of breast cancer treatment, ultimately improving patient care and outcomes.
